# Association Between The Real Cost Media Campaign and Smoking Initiation Among Youths — United States, 2014–2016

**DOI:** 10.15585/mmwr.mm6602a2

**Published:** 2017-01-20

**Authors:** Matthew C. Farrelly, Jennifer C. Duke, James Nonnemaker, Anna J. MacMonegle, Tesfa N. Alexander, Xiaoquan Zhao, Janine C. Delahanty, Pamela Rao, Jane A. Allen

**Affiliations:** ^1^RTI International, Research Triangle Park, North Carolina; ^2^Center for Tobacco Products, Food and Drug Administration, Silver Spring, Maryland; ^3^Department of Communication, George Mason University, Fairfax, Virginia; ^4^Akira Technologies, Washington, D.C.

In the United States, approximately 900,000 youths smoke their first cigarette each year (*1*). Health communication interventions are evidence-based strategies for preventing the initiation of tobacco use, promoting and facilitating cessation, and changing beliefs and attitudes about tobacco use (*2*,*3*). This report describes the association between the Food and Drug Administration’s (FDA’s) first national tobacco public education campaign, The Real Cost, and rates of smoking initiation among youths in the United States from 2014 to 2016. A nationally representative cohort study of youths (N = 5,185) was conducted during November 2013–March 2016. Results from a discrete-time survival model indicate that, among youths who reported never having smoked a cigarette in the baseline survey, the odds of reporting smoking initiation at follow-up were lower among youths with frequent exposure to campaign advertisements than among those with little or no exposure (adjusted odds ratio [aOR] = 0.70, 95% confidence interval [CI] = 0.55–0.91). Based on the results of the model, The Real Cost is associated with an estimated 348,398 U.S. youths aged 11–18 years who did not initiate smoking during February 2014–March 2016. Sustained youth-focused tobacco education campaigns, such as The Real Cost, can help speed progress toward preventing tobacco use among youths in the United States.

FDA’s The Real Cost was based on behavior change theories and designed to prevent the initiation of cigarette smoking among youths who have never smoked and discourage further smoking among youths who have previously experimented with smoking (*4*) (RTI International and FDA, unpublished data, 2016). Since February 2014, the campaign has aired tobacco education advertising designed for youths aged 12–17 years on national television, radio, the Internet, and in out-of-home displays, as well as in magazines and movie theaters (*4*). The central theme of the campaign is “Every cigarette costs you something.” In the first 3 years of advertising, campaign themes focused on the cosmetic effects of smoking, loss of control caused by addiction, and the dangerous mix of toxic chemicals in cigarette smoke.* To monitor campaign awareness levels (*4*) and evaluate the impact of The Real Cost on changes in smoking-related beliefs (RTI International and FDA, unpublished data, 2016) and behaviors, FDA conducted a national representative cohort study of U.S. youths. Youths aged 11–16 years at baseline were randomly selected from within 75 U.S. media markets and, after obtaining parental permission and youth assent, were interviewed in person at baseline during November 2013–March 2014. Data collections for the three follow-up surveys were conducted during July–October 2014, April–July 2015, and December 2015–March 2016 and consisted of online or in-person interviews.^†^ This report used data from the baseline survey and the first three follow-up surveys to determine whether campaign exposure was associated with preventing smoking initiation among youths who had never smoked at baseline (never smokers). The analytic sample consisted of 5,185 eligible youths, and the model included 11,145 observations across the surveys.^§^

Self-reported campaign media exposure was assessed with a validated measure (*5*) at each follow-up survey via video stream embedded within the survey. After viewing each advertisement, respondents reported their frequency of exposure to the advertisement on a scale from 0 (never) to 4 (very often). Respondents viewed all advertisements airing during the 3 months preceding each follow-up survey (a total of four advertisements at first and second follow-ups, and six advertisements at third follow-up). The frequency of exposure to all ads in each survey were summed, resulting in a score ranging from 0 to 16 at first and second follow-ups and from 0 to 24 at third follow-up. A dichotomous exposure measure was then created, defined as either low campaign exposure (<4) or high campaign exposure (≥4). Smoking initiation was defined as first trial of a cigarette among youths who had never used cigarettes.^¶^

A discrete-time survival model (*6*,*7*) was estimated using logistic regression and controlling for confounding influences, similar to other longitudinal media studies (*8*).** Because the delivery of advertisements is not explicitly random, the model included four types of potential confounders: demographic characteristics, individual risk factors for smoking cigarettes, self-reported exposure to other national campaigns (CDC’s Tips From Former Smokers and Truth Initiative’s truth campaign), and media market and state-level variables. The estimated number of youths prevented from initiating smoking was calculated using the difference between the predicted risk for initiation by age with actual exposure to The Real Cost campaign and the predicted risk for initiation by age in a hypothetical scenario where self-reported exposure to the campaign is either absent or low nationwide. The difference in initiation rates was then applied to the national population of nonsmoking youths at each age (2010 Census), and the resulting estimated numbers of youths potentially prevented from initiating smoking at each age were summed. Sensitivity analyses were conducted to examine the influence of electronic cigarettes (e-cigarettes) and other tobacco products on smoking initiation. An additional model examined the relationship between campaign exposure and using marijuana, a risky behavior unrelated to campaign messaging. This additional analysis was conducted to ascertain whether campaign effects were specific to smoking behaviors and not a general association between campaign exposure and risky behaviors.

High campaign exposure was associated with a 30% decrease in the risk for smoking initiation (aOR = 0.70, 95% CI = 0.55–0.91) (Table). The decrease in the risk for smoking initiation is illustrated by the difference between the risk for initiation with actual exposure to The Real Cost and the risk for initiation in a hypothetical scenario where there is no or low self-reported exposure to The Real Cost nationwide (Figure 1). Based on the results of the survival model, an estimated 348,498 youths aged 11–18 years were potentially prevented from initiating smoking nationwide during February 2014–March 2016 (95% CI = 331,825–365,168) (Figure 2).

**TABLE:** Results of a discrete-time survival model of the relationship between self-reported exposure to The Real Cost media campaign and smoking initiation by youths aged 11–18 years — United States, 2014–2016

Explanatory variable*	OR (95% CI)
**High exposure to The Real Cost (referent = no or low exposure)**	0.70^†^ (0.55–0.91)
**Gender (referent = female)**	
Male	1.03 (0.86–1.24)
**Race/Ethnicity (referent = white, non-Hispanic)**	
Black, non-Hispanic	1.35 (0.99–1.84)
Hispanic	1.39^†^ (1.11–1.73)
Other, non-Hispanic	0.77 (0.54–1.09)
**Youth income** ^§^	1.03 (0.99–1.07)
**Lives with tobacco user^¶^**	2.44** (2.04–2.92)
**Sensation seeking scale^††^**	1.40** (1.25–1.56)
**School environment^§§^**	0.85^†^ (0.77–0.94)
**School performance^¶¶^**	0.78** (0.70–0.87)
Educational plans***	0.92^†††^ (0.84–1.00)
**Parental communication^§§§^**	0.84** (0.76–0.94)
**Television use^¶¶¶^**	1.03^†^ (1.01–1.06)

**Abbreviations:** CI = confidence interval; OR = odds ratio.

*Additional control variables include average market-level family income, average market-level high school completion rates, market population, 2013 Behavioral Risk Factor Surveillance System state smoking prevalence, measures of self-reported exposure to the Tips From Former Smokers and the Truth Initiative’s truth campaigns, an indicator for whether the youth’s baseline interview was conducted after the launch of The Real Cost, age indicators, and time trend indicators.

^†^ p<0.01.

^§^ The amount of weekly discretionary income.

^¶^ Lives with a person who uses tobacco, including cigarettes, cigars, hookah, smokeless, and other tobacco products.

** p<0.001.

^††^ The brief sensation seeking scale (BSSS-4) is a mean of four items: 1) “I would like to explore strange places”; 2) “I like to do frightening things”; 3) “I like new and exciting experiences, even if I have to break the rules”; and 4) “I prefer friends who are exciting and unpredictable.” Responses ranged from 1 (disagree strongly) to 5 (agree strongly).

^§§^ School environment was measured as the mean of three items: 1) “I feel close to people at my school”; 2) “I am happy to be at my school”; and 3) “I feel like I am a part of my school.” Responses ranged from 1 (disagree strongly) to 5 (agree strongly).

^¶¶^ School performance was assessed with the item “How well would you say you have done in school?” with response options from 1 (much worse than average) to 5 (much better than average).

*** School aspirations were assessed with the item “How far do you think you will go in school?” with response options from 1 (I don’t plan to go to school anymore) to 8 (graduate, medical, or law school).

^†††^ p<0.05.

^§§§^ A youth’s relationship with parents was a mean of two items: 1) “Thinking about the adult or adults you live with would you say you are satisfied with the way you communicate with each other” (responses from 1 [very unsatisfied] to 5 [very satisfied]), and 2) “How close do you feel to the adult or adults you live with?” (Responses ranged from 1 [not close at all] to 5 [very close]).

^¶¶¶^ Continuous variable of daily hours spent watching television across all media devices.

**FIGURE 1:**
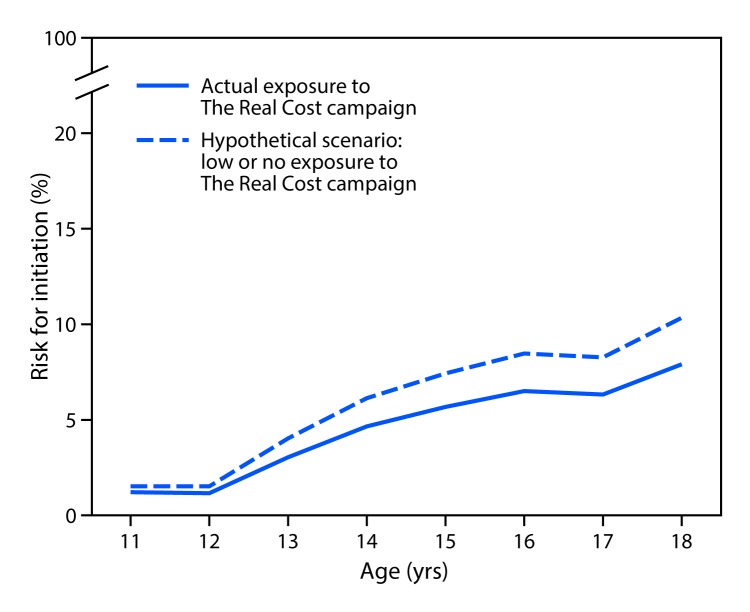
Estimated smoking initiation risk among youths aged 11–18 years with actual exposure versus hypothetical scenario with low or no exposure to The Real Cost campaign, by age — United States, 2014–2016

**FIGURE 2:**
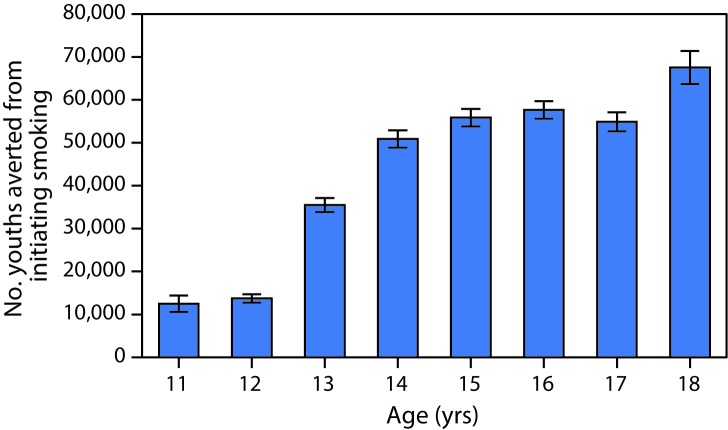
Predicted number of youths* aged 11–18 years potentially prevented from initiating smoking as a result of The Real Cost campaign, by age — United States, 2014–2016 * With 95% confidence intervals represented by error bars.

The association between campaign exposure and youth smoking initiation remained unchanged in survival models that accounted for youths’ use of e-cigarettes and other tobacco products during the study period. In a similar survival model, exposure to The Real Cost was not associated with a change in the likelihood of marijuana initiation.

## Discussion

The findings from this analysis indicate that exposure to The Real Cost campaign was associated with preventing an estimated 348,398 U.S. youths aged 11–18 years from initiating smoking during 2014–2016. Most tobacco dependence begins during adolescence (*3*), and youth-focused campaigns to prevent smoking initiation, such as The Real Cost, can have long-term effects on future rates of tobacco-related morbidity and mortality (*9*).

Findings from this report support previous studies that indicate The Real Cost meets or exceeds guidelines for effective health communication interventions (*2*). FDA conducted formative research to develop campaign advertisements for The Real Cost, including qualitative and quantitative testing of campaign messages and draft advertisements (RTI International and FDA, unpublished data, 2016). Since its launch, campaign advertising has occurred with high frequency across multiple media channels targeting youths. Research indicates that approximately 9 of 10 youths reported seeing The Real Cost advertisements after 7 months, with more youths reporting awareness of advertising in subsequent surveys (*4*). The Real Cost was also found to positively influence tobacco-related risk perceptions and beliefs specific to campaign messages after 15 months (RTI International and FDA, unpublished data, 2016). These results demonstrate the effectiveness of a national campaign that focused on the harmful effects of smoking and delivered salient messages that resonated with youths.

These findings align with previous research that found targeted mass media campaigns, delivered with sufficient intensity and duration, can decrease smoking initiation and prevalence (*2*,*9*). A comprehensive tobacco control approach that emphasizes proven strategies, such as The Real Cost, can result in reductions in smoking among youths today, and such reductions can lead to decreased future rates of smoking-attributable mortality, health care costs, and lost workplace productivity (*3*,*9*).

The findings in this report are subject to at least four limitations. First, measurements were self-reported and are subject to bias. Specifically, selective attention could bias the results, such that nonsmoking youths at risk for future smoking might be more likely to both pay attention to campaign messages and experiment with smoking. However, such a positive association would be expected to lead to smaller observed campaign effects on initiation. In addition, social desirability bias might have led to underreporting of initiation and overreporting of campaign exposure. To address the concerns of using self-reported exposure, future research that examines potential campaign exposure based on measures of market-level media delivery (i.e., target rating points^††^) is warranted. Second, although the model controls for youths’ exposure to other tobacco-related media campaigns, this might not fully account for the independent or synergistic effects of the other campaigns. Third, sample attrition might result in bias. However, attrition analyses indicate the baseline and follow-up samples were similar across demographics, susceptibility to smoking cigarettes, and household tobacco use. Finally, because of sample size limitations, only initiation to smoking was examined, not progression to established or daily smoking. Future analyses could examine the campaign’s effect on youth smoking prevalence and further explore the campaign’s effect among demographic subgroups.

The Real Cost is the first federally funded U.S. youth-focused tobacco education campaign, and these findings indicate that youths’ self-reported exposure to the campaign was associated with a reduction in smoking initiation during the evaluation’s 2014 to 2016 time frame. Sustained tobacco education campaigns such as The Real Cost can encourage U.S. youths to abstain from tobacco use and accelerate progress toward future tobacco-free generations.

**Summary**

**What is already known about this topic?**

Public education campaigns are evidence-based strategies for positive public health outcomes such as preventing the initiation of tobacco use, promoting and facilitating cessation, and shaping social norms related to tobacco use.

**What is added by this report?**

This study is the first to examine the association between the Food and Drug Administration’s (FDA’s) youth-specific national media campaign, The Real Cost, and adolescent smoking in the United States. Approximately 350,000 youths aged 11–18 years were prevented from smoking nationwide during 2014–2016 as a result of FDA’s youth-specific public education campaign.

**What are the implications for public health practice?**

The findings indicate that youths’ self-reported exposure to the campaign was associated with a reduction in smoking initiation from 2014 to 2016. Sustained campaigns such as The Real Cost can speed progress toward a tobacco-free future.

## Footnotes

* http://www.fda.gov/TobaccoProducts/PublicHealthEducation/PublicEducationCampaigns/TheRealCostCampaign/.

† All youths aged 11–16 years in selected households were eligible for the baseline survey. Youths were selected within 75 Nielsen Designated Market Areas using 2010 Census Bureau block groups as the secondary sampling unit. At baseline, the unweighted household-level response rate was 48%, and follow-up response rates ranged from 87% to 91% (American Association of Public Opinion Research Response Rate #3 formula).

§ The analytic sample included youths who reported they had never smoked (never smokers) at baseline and completed at least one follow-up survey (91% of respondents were never smokers at baseline and of these, 87% responded to at least one follow-up survey).

¶ “Never use” of cigarettes was examined using the measure: “Have you ever tried cigarette smoking, even one or two puffs?” including response options of “Yes,” “No,” and “Don’t Know.” Youths who had never used cigarettes were coded as respondents who said “No.”

** This analytic approach begins with all youths who never smoked and then estimates the risk for smoking initiation as they age. Once the event of interest (initiation of smoking) occurs, the youth is dropped from subsequent time periods, thus allowing calculation of the probability that a youth will initiate smoking at each age, given that the youth had not previously begun smoking.

†† Nielsen's system of target rating points are the standard unit of measurement for media delivery and measure the reach and frequency of an advertisement among a target population.
